# Transposon and Transgene Tribulations in Mosquitoes: A Perspective of piRNA Proportions

**DOI:** 10.3390/dna4020006

**Published:** 2024-03-30

**Authors:** Nelson C. Lau, Vanessa M. Macias

**Affiliations:** 1 Department of Biochemistry and Cell Biology, Boston University Chobanian and Avedisian School of Medicine, Boston, MA 02118, USA; 2 Genome Science Institute and National Emerging Infectious Disease Laboratory, Boston University Chobanian and Avedisian School of Medicine, Boston, MA 02118, USA; 3 Department of Biology, University of North Texas, Denton, TX 76205, USA; 4 Advanced Environmental Research Institute, University of North Texas, Denton, TX 76205, USA

**Keywords:** transgene DNA, mosquito, small RNA silencing, Piwi, piRNAs

## Abstract

Mosquitoes, like *Drosophila*, are dipterans, the order of “true flies” characterized by a single set of two wings. *Drosophila* are prime model organisms for biomedical research, while mosquito researchers struggle to establish robust molecular biology in these that are arguably the most dangerous vectors of human pathogens. Both insects utilize the RNA interference (RNAi) pathway to generate small RNAs to silence transposons and viruses, yet details are emerging that several RNAi features are unique to each insect family, such as how culicine mosquitoes have evolved extreme genomic feature differences connected to their unique RNAi features. A major technical difference in the molecular genetic studies of these insects is that generating stable transgenic animals are routine in *Drosophila* but still variable in stability in mosquitoes, despite genomic DNA-editing advances. By comparing and contrasting the differences in the RNAi pathways of *Drosophila* and mosquitoes, in this review we propose a hypothesis that transgene DNAs are possibly more intensely targeted by mosquito RNAi pathways and chromatin regulatory pathways than in *Drosophila*. We review the latest findings on mosquito RNAi pathways, which are still much less well understood than in *Drosophila*, and we speculate that deeper study into how mosquitoes modulate transposons and viruses with Piwi-interacting RNAs (piRNAs) will yield clues to improving transgene DNA expression stability in transgenic mosquitoes.

## 1. Why Think of Fruit Flies When Mosquitoes Are Humanity’s Bigger Problem?

The fruit fly, *Drosophila melanogaster*, is an insect model organism that has had a huge impact on biomedical research, attributable to at least five Nobel prizes in Physiology and Medicine from 1933 to 2017 for discoveries of DNA chromosomes, DNA mutations, embryonic development, Toll receptors and circadian rhythms as well as numerous other breakthroughs [1–5]. Mosquitoes have also had a huge impact on humanity but as a major scourge of diseases for millennia, well before *Drosophila* researchers started pushing flies around in the lab in the 20th century.

Initial molecular work in mosquitoes sought to leverage the similarities between mosquitoes and their Dipteran cousins to apply advancements in genetics and address questions in vector-borne disease biology [6–8]. Both insects are in the order Diptera, having two wings, similar overall body plans and relatively short holometabolous life cycles with eggs, four larval instars, pupae and adults. At the cellular level, fruit flies and mosquito share many orthologous genes and similar cell types [9–11].

Beyond morphological and genetic similarities, biological differences set these two insects apart. Mosquitoes are one of only 17 taxa of insects to require blood as a nutrient source for reproduction [12]. This blood-feeding behavior is accompanied by remarkable biological specializations, i.e., anesthetizing piercing mouthparts, human-targeted saliva chemistry and extra innate immune functions. Because a female mosquito can take multiple blood meals in her lifespan, she can ingest, host and transmit pathogens to vertebrates, which elevates mosquitoes’ importance to agricultural and medical concerns. For additional background on the biological distinctions and comparisons between *Drosophila* fruit flies and mosquitoes, we refer readers to these reviews [13,14].

Despite a large community of mosquito researchers trying to manipulate mosquitoes over four decades, the molecular genetics of mosquitoes still lags behind *Drosophila. Drosophila* transgenesis has been routine for many decades to yield a multitude of stable transgenic stocks, yet stable transgenic mosquitoes are still an aspiration in this field that has benefitted from major advances in deep sequencing and genome editing.

In this review, we will discuss the differences in transgenesis effectiveness between fruit flies and mosquitoes and propose the hypothesis that natural gene regulatory responses in mosquitoes may be silencing synthetic transgenes. We suspect that a more complete understanding of the expanded RNA interference (RNAi) pathways in mosquitoes will enlighten avenues to side-step this. This important technical hurdle with transgenes continues to vex mosquito researchers who are envious of their fly-pushing colleagues and motivates us to discuss the major knowledge gaps in mosquito Piwi pathway biology.

Early transgenesis approaches in other organisms were well known to trigger gene silencing via RNAi which has been reviewed most recently in [15–17]. But well before RNAi was discovered in nematodes [18,19], plant biologists first observed the phenomenon of co-suppression more than 30 years ago in petunias where the unregulated integration of pigment-producing transgenes caused counterintuitive silencing of the endogenous pigment genes [20–22]. The concatamerization of many transgenes in plants led to the generation of double-stranded RNAs (dsRNAs) that subsequently yielded small interfering RNAs (siRNAs) with sequence homology to the transgene. Co-suppression in *Drosophila* was then discovered by the Birchler lab whereby excessive copies of a *white-Adh* transgene triggered silencing of the transgene and then the endogenous *Adh* gene [23]. Subsequent studies confirmed co-suppression phenomena in *Drosophila* between the triggering transgene and RNAi mechanisms that include transposon regulation [24,25].

To avoid co-suppression in *Drosophila*, modern transgene DNAs are integrated in low copy numbers via either short pulses of transposons like *P*-elements and *Minos* elements transposases, bacterial integrases like PhiC31, or now with CRISPR/Cas9-mediate genome editing [26–35]. Frequently, a visual genetic marker for positive selection in the transgenes facilitates the recovery of successful transformants even if transgenesis rates are a minor fractional yield [27,28,30,32]. The binary control of the GAL4-UAS transgene system in *Drosophila* further reduces the chance for ectopic misregulation of the transgene to trigger RNAi silencing [36,37]. Nevertheless, transgenes can be used to express miRNAs and siRNAs for RNAi- mediated gene silencing in *Drosophila*, as demonstrated by the genomewide genetic RNAi tools for knockdown studies [24,38–40].

Mosquito researchers have kept a close eye on *Drosophila* transgenesis approaches, and have adapted many similar DNA construct designs, transposons and embryo injection techniques used by *Drosophila* researchers [41,42]. Mosquito husbandry is more complicated than for *Drosophila*, but not so much as to inhibit obtaining first generation transgenic animals [43–48]. *Drosophila* benefit from a wider variety of visible genetic markers, much simpler husbandry requirements, broader mutant and transgenic stock collections and powerful balancer chromosomes that make *Drosophila* an unrivaled model organism for genetics studies [49–52].

Genetic innovations like binary promoter transgenes in mosquitoes could enable finer tuning of transgene expression to temper the triggering of mosquito RNAi and piRNA pathways. The *Drosophila* binary GAL4-UAS system is famous for temporal and tissue-specific regulation of transgene DNA expression with continuing innovation in this genetic technology [37,50,53]. Some groups have ported the binary GAL4-UAS system to *Aedes* and *Anopheles* mosquito strains [54–57], but the pace of deploying these transgenic systems broadly to the rest of the mosquito research community has not been rapid. Rather, a new binary transgenic platform called the Q-system from the Potter lab was first developed in *Drosophila* to be orthogonal to the GAL4-UAS system and [58,59] has now been successfully applied to *An. gambiae* [60] and *An. stephensi* transgenes [61].

All the advantages of the *Drosophila* methods need to be further developed for mosquito research, and the injection procedures with mosquitoes are more demanding; depending on the species, ~1500 mosquito embryos can be injected over two weeks to generate ~150 G0 offspring where ~2% of G1 this progeny will exhibit transgene expression (reviewed in [48,62]). The efficiency rates in *Drosophila* are an order of a magnitude higher and accessible enough that multiple commercial vendors provide transgenesis services (i.e., Rainbow Transgenic Flies, BestGene, WellGenetics, GenetiVision and CD BioSciences, to name a few).

The animal RNAi pathway generates siRNAs and microRNAs (miRNAs) that are the most broadly expressed small RNAs bound by Argonaute proteins. Unique to RNAi in animals is a third type of small RNA bound by Piwi protein called Piwi-interacting RNAs (piRNAs) first discovered in *Drosophila* to have a function in silencing transposons in the germline to ensure female fertility [63–67]. Several reviews cover the history and recent discoveries of the piRNA pathway in *Drosophila* [16,68–70]. For this perspective, focusing on the problem with mosquito transgenesis, we will highlight specific features of the *Drosophila* piRNA pathway that may inform regarding the mosquito piRNA pathway reacting adversely to transgenes.

## 2. The Challenges in Stable Transgene Expression in Mosquitoes

**Figure 1.**
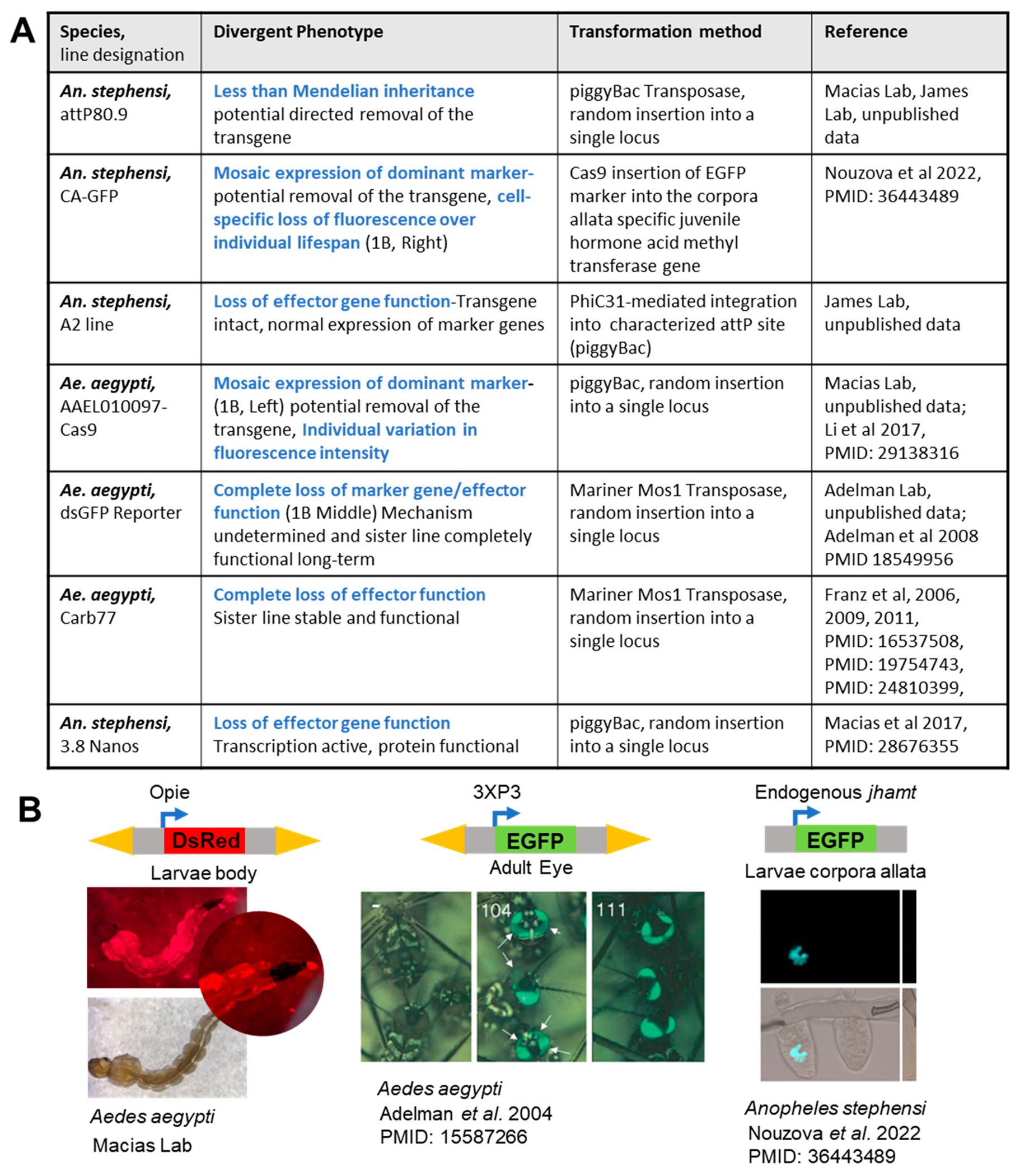
Tribulations with transgenes in mosquitoes. (**A**) A table that includes previously published and personal communications from unpublished results of anomalous transgene activity in mosquitoes [43–48,79]. (**B**) Mosaic expression of the marker gene in examples from some of the experiments listed in (**A**) [74,79]. Loss of marker expression in a patchy distribution has been identified in several transgenes and is indicative of different transgene activity and/or repression in different cell groups. jhamt: juvenile hormone acid methyl transferase.

Although stable transgenic strains were already a mainstay of *Drosophila* genetics over 30 years ago, the history of transgenesis in mosquitoes is fraught with unresolved setbacks despite recent advances in mosquito genome editing with new CRISPR-Cas9 methodologies [43,71–73]. In Figure 1A, we summarize transgenesis tribulations in mosquitoes to bring to light both documented and anecdotal evidence that the transgenesis challenge remains in the field of mosquito genetics.

The literature has documented at least three transgenic *Aedes aegypti* mosquito lines with anomalies in marker expression, anti-pathogen effector activity and gene-drive expression stability. In a 2004 report, one of several *Ae. aegypti* strains transformed with the same EGFP-marker showed mosaic expression of the eye marker (Figure 1A and Figure 1B middle panel) and resisted careful crossing to a homozygous state [74], perhaps reflecting other reports of *Ae. aegypti* strains that succumb to attempts at inbreeding to an isogenic state [45,75–78]. An *Ae. aegypti* line transformed to express GFP under an intrinsic promoter specific to the corpora allata was inconsistently expressed and erratically regulated, showing variable expression of the marker within the tissue (Figure 1A and Figure 1B right panel) [79].

Lastly, transgene suppression can occur sporadically over the course of multiple breeding generations, as shown by the sudden suppression of an inverted repeat transgene to make dsRNA against dengue virus in the *Ae. aegypti* Carb77 line [45–47]. Although the antiviral transgene was initially stably expressed, antiviral suppression by the transgene subsided in the 14th generation and was completely silent by the 17th generation without any mutations to the transgene [46].

The malaria-vectoring genus, *Anopheles*, also has documented problems with transgene stability. An *An. stephensi* piggyBac-mediated gene drive element designed to mobilize a synthetic transgene could only be detected to move about once every 20 generations, though the piggyBac transposase was highly effective when provided in trans in this species [48]. Another cautionary tale is with a transgenic *An. gambiae* strain Ag(PMB)1 whose transgene was engineered to express an endonuclease designed to cause sex-distortion [80]. In this transgenic *An. gambiae*, the precise genomic integration site of the piggyBac transgene could not be determined despite extensive high-throughput whole genome sequencing and fluorescent in situ hybridization [81]. The only hint of the transgene DNA integration site was highly repetitive flanking sequences with homology to centromeric regions of the *An. gambiae* genome.

In addition to the published reports above, there are anecdotes of transgenic mosquito lines that ultimately were discarded because of malfunction with phenotypes deviating from the desired regulation of the transgene. In just two years, two transgenic lines under our care have ceased to function properly. For instance, larvae in an *Ae. aegypti* transgenic line-*AAEL010097*-Cas9 that was generously gifted to the Macias lab by Dr. Akbari’s group, first start out with robust body expression of the DsRed fluorescence marker (Figure 1B, left). After ~16 generations, some individuals began to exhibit a patchy body expression in one family, while a sister family remains normally expressing to this day.

A second instance is one of several *An. stephensi* lines with the same PhiC31 docking site insert, a line designated attP80.9, that did not follow Mendelian inheritance, suggesting removal of the transgene from some individuals. This is strikingly similar to the first GFP-expressing line in *Ae. aegypti* mentioned above, and this transgene’s DNA may also be flanked by repetitive viral sequences [74]. We proposed that the loss of the transgenes at each generation in our Anste80.9 line could be attributed to the transgene being inserted as tandem repeats into a repetitive region of ribosomal RNA genes clusters (rDNA), which in *Drosophila* has wide flux in genomic copy number including gene copy loss and is subject to retrotransposon regulation [82–86]. Given that two independent *Anopheles* lines and an *Aedes* line all have integrated the transgenes into repetitive loci raises the specter of RNAi silencing because many *Drosophila* siRNAs and piRNAs derive from transposon-rich sequences including pericentromeric and rDNA loci [66,86,87].

In summary, the phenotypes of the instability of mosquito transgenes described here falls into these categories: (1) rapid loss of effector/marker function, (2) mosaic expression of gene function within individuals and (3) loss of the transgene over generations. Could these undesired phenotypes reflect different mechanisms of recognition and silencing by the mosquito piRNA pathways?

## 3. Recapping What Makes Mosquito piRNA Pathways Special

We have recently reviewed the general features of mosquito Piwi pathways and piRNAs and the importance of this biology to mosquito genetics and vector biology in [13,14]. As we consider the hypothesis proposed above that mosquito piRNA pathways may impinge upon transgene expression, we will recap additional salient features of the piRNA pathways in *Drosophila* and mosquitoes. Transposon silencing by Piwi proteins and piRNAs is a conserved function across animals, as reflected by increased transposon RNAs and transposon copy number expansion in gonads from Piwi pathway loss-of-function mutants in mouse and fruit flies [16,68,69,88]. Although Piwi gene mutants in mosquitoes have not yet been generated and studied for transposon silencing and fertility roles, knockdowns of Piwi genes in mosquito cells [89–94], and inhibitions of specific mosquito piRNAs can lead to upregulation of transposon mRNAs [95,96].

**Figure 2.**
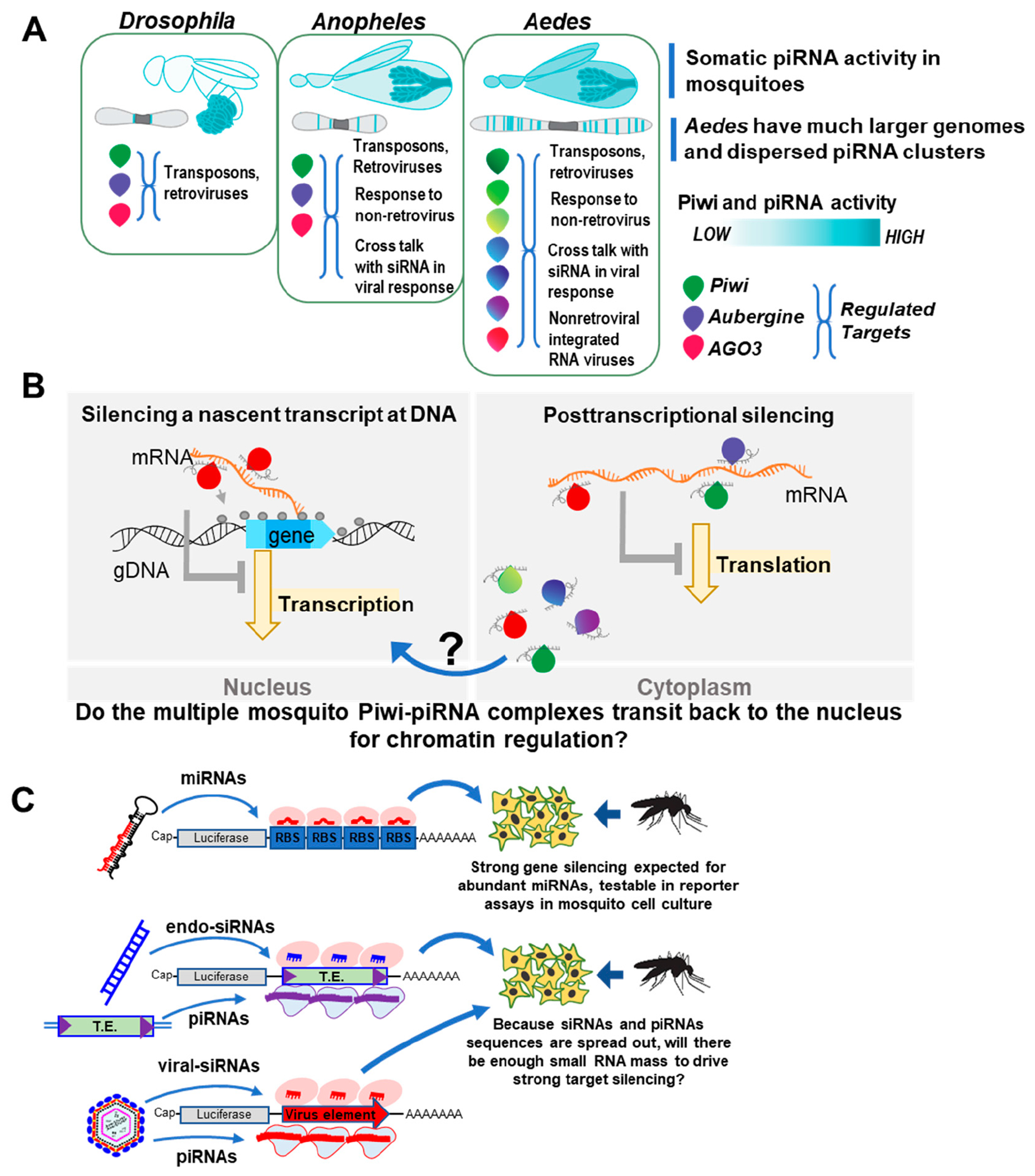
Comparing Piwi-piRNA pathways between *Drosophila* and mosquitoes and testing for piRNA silencing capacity. (**A**) Diagram highlighting the major somatic Piwi and piRNA activity in mosquitoes, the expanded genome size in *Aedes* mosquitoes, and the expanded DNA copies of Piwi gene homologs. (**B**) Simplified diagram of the nuclear and cytoplasmic mechanisms of piwi/piRNA target silencing in *Drosophila* germ cells and gonads, and the open questions towards where mosquito Piwi/piRNA complexes are partitioned. (**C**) Diagram illustrating the design of DNA reporter assays needed to measure the small RNA silencing capacities at a biochemical level to compare mosquito miRNAs to siRNAs and piRNAs.

Mosquitoes may have a specialized piRNA pathway that is more active against other genetic intruders beyond transposons, such as targeting several classes of insect specific viruses as well as arboviruses [90,97–102]. Although anopheline mosquitoes have the same number of Piwi-protein genes as *Drosophila*, culicine mosquitoes have expanded the number of Piwi-protein genes and representation of piRNA clusters on the genome (Figure 2A). Anopheline mosquitoes are more prolific at vectoring malaria-causing parasites and only a few viruses, whereas culicine mosquitoes (*Culex* and *Aedes* genera) are the main transmitters of arthropod-borne viruses like dengue, chikungunya and Zika viruses [13]. It is still unclear if the differences between anopheline and culicine mosquitoes in hosting pathogens could be related to the piRNA and Piwi pathway differences between these two mosquito classes. Nevertheless, we will next discuss three notable features of the Piwi pathway particularly with culicine mosquitoes.

The first notable feature of culicine mosquito piRNA clusters is the greater frequency of RNA virus genomic sequences being integrated into these loci to generate Endogenous Viral Element (EVE) piRNAs. For example, a subset of piRNA clusters in *Ae. aegypti* and *Ae. albopictus* is enriched with fragments of various single-stranded RNA virus sequences [103–106]. Since these are not retroviruses that would typically retrotranscribe into DNA and integrate into the host genome, the mechanism is still unclear for how *Aedes* piRNA clusters can trap these viral sequences, but perhaps it reflects these viruses being vectored more frequently in culicine mosquitoes versus anopheline mosquitoes. Additionally, there is crosstalk between virus infection and host piRNA expression, as was observed by chikungunya virus infections of *Ae. albopictus* [107] and Zika virus infection of *Ae. aegypti* [97] stimulating changes in the endogenous piRNAs in these two mosquito species.

The second notable feature is that while *Drosophila* (and most vertebrates) only express Piwi proteins and piRNAs abundantly in the gonads, mosquitoes also abundantly express Piwi proteins and piRNAs in almost the entire somatic body like in most other arthropods [97,108,109]. There is still abundant Piwi proteins and piRNAs in mosquito gonads to prevent germline transposition of transposons and ensure fertility. However, it is not clear how effective are the Piwi/piRNA complexes at suppressing transposons and insect viruses since transposons and viruses are persisting and expanding in culicine mosquitoes [97,103,110]. We anticipate that there will be important biological differences between the somatic and germline Piwi pathway components within mosquitoes, but the somatic Piwi pathway components will be easier to conduct biochemical studies due to many of the mosquito cell lines derived from embryo and larvae cells of somatic origin [97].

The third notable feature of culicine mosquito piRNA pathways is the expansion of the Piwi protein gene family to seven Piwi genes in contrast to just three Piwi members in *Drosophila* and anopheline mosquitoes (Figure 2A) [13,97,111]. One possible rationale for this gene family expansion is for culicine mosquitoes to evolve specialized silencing functions like AGO3, PIWI5 and PIWI4 preferentially interacting with viruses in infected cells and primarily loading piRNAs antisense to viral genomes [92,93,102,112,113]. Another rationale is for culicine mosquitoes to generate an expanded range of piRNAs from transposons, viruses and protein coding genes, but still have piRNA biogenesis patterns via the ping-pong mechanism and 5′ end phasing be strongly enforced in discreet ways. For example, the 5’ end phasing periodicity of piRNAs in culicine mosquitoes is extremely regular [97,114], much more so than in *Drosophila*, and this may be aided by novel mosquito protein factors orchestrating this ping-pong activity in piRNA biogenesis [90,94,114,115].

Much of our current understanding of mosquito piRNA targeting functions is limited to our genomics mapping of small RNAs to mosquito piRNA clusters, and our annotation of known invading elements like transposons and nrEVEs to these loci. With so much diversity in structural features of mosquito piRNAs, including novel satellite-like piRNAs in culicine mosquitoes [95,97,103,110], it is tempting to propose varied and nuanced silencing capacities amongst the many different types of piRNAs that could emanate from mosquito piRNA clusters. Indeed, we still do not fully understand the biological impact of each major piRNA cluster locus in mosquitoes, such as whether they can trap transgenes in cis or silence transgenes in trans from the millions of different individual piRNA sequences. Furthermore, if the only main function for transposon and viral piRNAs is gene silencing, we ponder why both invading elements continue to proliferate so widely in culicine mosquitoes.

However, to formally test the function of mosquito Piwi pathway genes and functions for each piRNA cluster, the mosquito field needs to generate loss-of-function mutants like in *Drosophila* and mice. With recent CRISPR advances for mosquitoes like the ReMOT technology [71,72], this goal is certainly within reach to generate loss of function mutants in mosquito Piwi pathway genes and piRNA clusters. Nevertheless, we should anticipate potential challenges in finding obvious phenotypes in mosquito individual piRNA cluster mutants since many major piRNA cluster mutants in fruit flies and mice were also superficially normal [116–119]. Pragmatically, a mosquito piRNA cluster mutant that is phenotypically normal but now has stable transgene expression would be a huge boon to the research community.

These notable Piwi pathway features in mosquitoes will need to be integrated into a better working model for how the mosquito Piwi pathway could be responding to unpredictable silencing of mosquito transgenes. Are transgenes instigating piRNAs like viruses and transposons? Are specific mosquito Piwi proteins the prime silencer of transgenes? Which compartments are more active at transgene silencing, the germline or the whole soma? Perhaps the major knowledge gap holding us up from developing this better integrated model for transgene silencing by mosquito piRNAs is the next question.

## 4. Nuclear versus Cytoplasmic Piwi-piRNA Complexes: What Do Mosquitoes Favor?

To appreciate how *Drosophila* piRNA pathways can instigate robust and heritable transposon silencing in the germline, we need to compare the nuclear and cytoplasmic activities of the *Drosophila* Piwi pathway and then extend this view to our current understanding of the mosquito Piwi pathway. Within the *Drosophila* ovary are two distinct piRNA pathways to control transposon activity, a somatic follicle-cell version with only a single PIWI protein and a nurse cell and oocyte version where AGO3 and AUBERGINE (AUB) coordinate with PIWI [66,87]. In nurse cells, AGO3 and AUB are localized in the cytoplasm in perinuclear condensates called “nuage” presumably targeting transposon mRNAs for destabilization or inhibiting translation [120–122]. PIWI is localized to the nucleus in both follicle cells and nurse cells, locating transposon nascent transcripts and recruiting a cascade of chromatin modifying proteins to engage in heterochromatin formation [123–128]. These reviews further cover the molecular details of the *Drosophila* piRNA transcriptional silencing mechanism [69,129].

Although the *Drosophila* oocyte is mainly inactive in transcription, it gains massive maternal contribution of proteins and RNAs from the surrounding nurse cells, including the Piwi/piRNA complexes [130–134]. Furthermore, biochemical communication is required between the cytoplasmic AGO3 and AUB and the nuclear PIWI to promote piRNA amplification and facilitating this maternal contribution from nurse cells to oocytes [124,135]. Although maternal contribution of follicle cell PIWI/piRNA complexes is unlikely, there are endogenous retrovirus particles that can emerge from follicle cells to infect the oocyte, and this is suppressed by the PIWI/piRNA complex in these follicle cells [136–140].

The staggering complexity of >40 factors in the *Drosophila* Piwi pathway to engage in piRNA biogenesis and transposon silencing at transcriptional and post-transcriptional levels is a testament to the essentiality of suppressing transposons to protect the germline genome’s integrity. New factors that contribute to this piRNA-triggered heterochromatin formation are still being uncovered in *Drosophila* genetic studies [83,130,141–143].

The majority of *Drosophila* Piwi pathway factors are conserved in mosquito genomes, but key nuclear factors that enable *Drosophila* PIWI to trigger transcriptional silencing on transposon targets are notably missing in mosquito genomes. *Drosophila* PANX, NXF2 and NXT1 comprise the SFiNX-PICTS-PPNP-Pandas complex that connects PIWI to Su(var)2-10 and Eggless/SETDB1 to trigger transcriptional silencing [142–149]. Although NXF2 and NXT1 are conserved in mosquitoes, the central factor PANX is absent in both anopheline and culicine mosquito genomes. The Rhino/Del/Cuff complex that also mediates dual strand piRNA cluster expression and transposon silencing [150–152] is also a *Drosophila*-specific set of genes with no clear orthologs in mosquitoes.

The transit and localization of the natural Piwi/piRNA complexes in mosquitoes is becoming clearer (Figure 2B) as some epitope-tagged cell culture-based studies and cell-fractionation experiments indicate mosquito PIWI4, PIWI5, PIWI6 and AGO3 are predominantly cytoplasmic with a minor fraction retained in the nucleus [90,94]. A second study focusing on just *Ae. aegypti* PIWI4 corroborated the endogenous pattern in midgut and ovary as mostly cytoplasmic and a minor fraction in the nucleus [153]. When additional antibodies to mosquito PIWI-2,-3, and −7 become available, or CRISPR gene editing can insert epitope tags in all endogenous mosquito Piwi genes, the field will have better tools to fully assess which cellular compartments contain mosquito Piwi/piRNA complexes.

Mass spectrometry of mosquito Piwi protein complexes have recently identified new mosquito-specific homologs of piRNA biogenesis factors primarily acting in the cytoplasm. Associating with Ae-AGO3 is Atari-PB, an *Aedes* ortholog of the *Drosophila* Tudor-domain protein Krimper [94]. Associating with the *Aedes* PIWI4, −5, and −6 is Pasilla (Ps) which in *Drosophila* is only a splicing factor but in *Aedes* there is a special cytoplasmic variant Ps-PB-PK that additionally promotes mosquito piRNA biogenesis [94]. Cytoplasmic granules of Atari-PB, Ps-PB-PK and another Tudor domain-containing protein in mosquitoes, Veneno [115], indicate that this aspect of mosquito piRNA biology follows the trend of protein–RNA condensates as piRNA biogenesis centers conserved in many other animal germ cells from invertebrates to vertebrates [121,122,128,154–159].

## 5. The Importance of Biochemical Silencing Capacity Assays for Mosquito Small RNAs

If transgene expression instability is caused by piRNA-targeted silencing, we need to determine how many piRNAs matching to transgenes from small RNA sequencing will trigger silencing, either through post-transcriptional cytoplasmic activity or transcriptional nuclear activity. Cytoplasmic activity is mainly understood around mosquito piRNA biogenesis mechanisms that follow the ping-pong amplification of piRNAs coupled with string of trailing responder piRNAs emanating from the initial trigger piRNA [91,94,98,99,114,115]. The verdict is still out for whether mosquito Piwi/piRNA complex can carry out transcriptional, heterochromatic silencing, but some reporter gene assays to measure mosquito piRNA silencing do hint to possible nuclear regulation of a single special piRNA targeting a reporter gene sequence within an intron [95].

We propose broadening the biochemical reporter gene assays from other metazoan studies to mosquitoes (Figure 2C) to determine how potent and stochiometric is gene silencing mediated by the different amounts of mosquito miRNAs, siRNAs and piRNAs. For example, in *Drosophila* OSS and OSC cells, luciferase and GFP reporter assays provided quantitative measure of gene silencing capacity between these small RNAs, indicating that *Drosophila* cells require many thousands of piRNA reads per million against a target transposon sequence to trigger silencing [144,160–162]. Because piRNA biogenesis patterns are a complicated mix of ping-pong interactions between trigger and responder piRNAs [163–167], most individual piRNA sequences in *Drosophila* are too low in abundance to trigger silencing like a miRNA.

**Figure 3.**
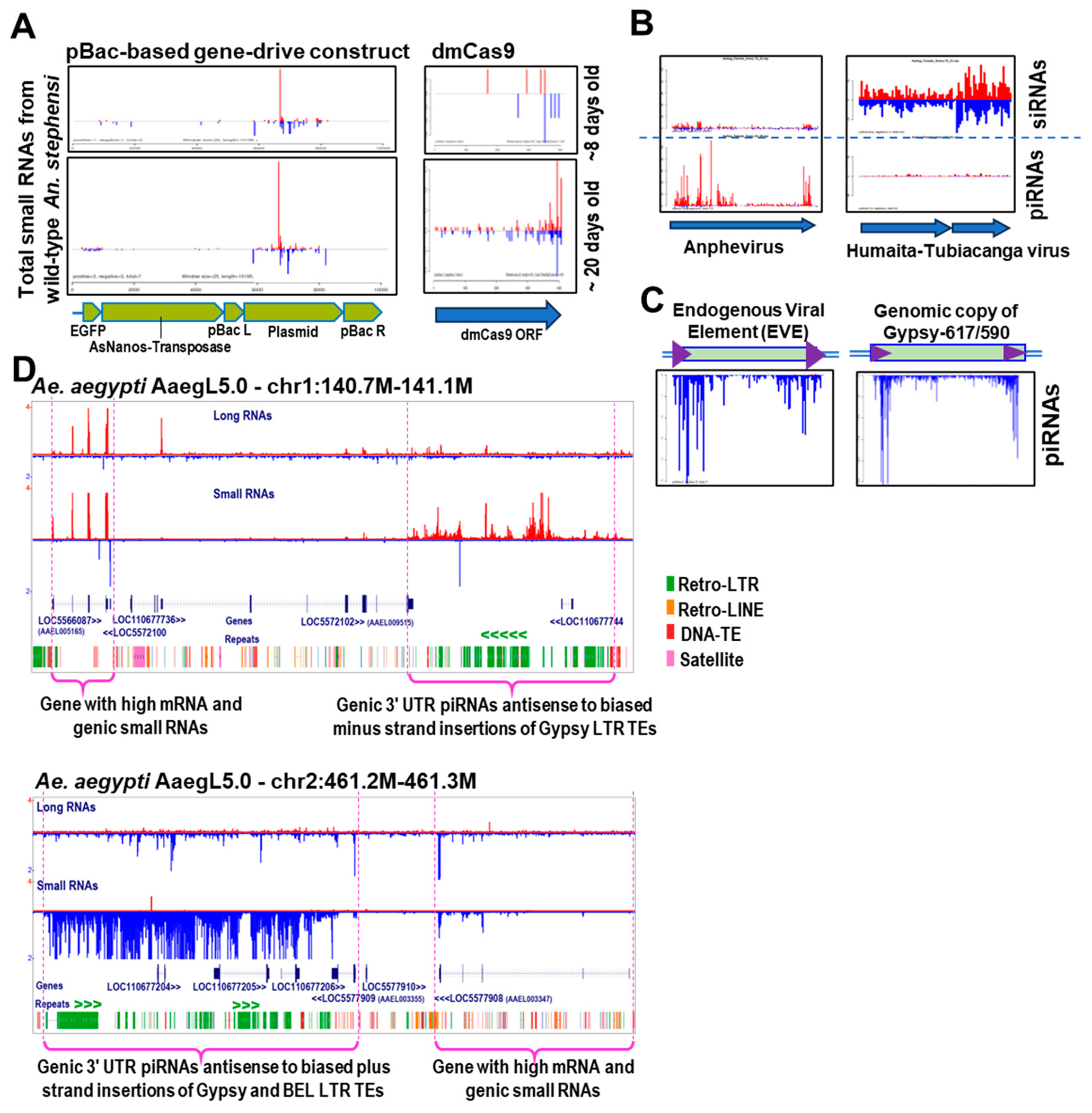
Endogenous mosquito small RNAs targeting transgenes silencing and originating from a myriad of piRNA precursor loci. (**A**) Examples of endogenous wild-type *Anopheles stephensi* small RNAs that have homology to a pBac-based gene-drive transgene DNA construct described in Macias et al., 2017 [48]; as well as against a *Drosophila*-codon-optimized Sp-Cas9 gene. Red bars are plus strand reads while blue bars are minus strand reads. The transgene-targeting piRNAs appear to accumulate more over time. Sequencing data from Henderson et al., 2022 [168]. (**B**) Two examples of viral small RNAs, and (**C**) a comparison of antisense piRNA patterns targeting an Endogenous Viral Element versus a *Gypsy* transposon consensus sequence. (**D**) Adapted UCSC Genome Browser windows of two major intergenic piRNA clusters from *Aedes aegypti* where the transposon insertions are biased to be antisense to the production of piRNAs. *Ae. aegypti* small RNA data from Ma et al., 2020 [97], analyzed through the Mosquito Small RNA Genomics resource.

In a preliminary search for evidence of transgene regulation by the piRNA pathway in existing *An. stephensi* small RNA libraries [168], we identified 24–30 nucleotide small RNAs with homology to commonly used transgene construct elements (Figure 3A). However, such data is currently challenging to interpret without a test in mosquitoes of whether a modest number of piRNAs can trigger silencing, or if a bulk amount of piRNAs like from certain genic piRNA clusters in mosquitoes are required to trigger silencing like in *Drosophila* cells [144,160–162].

Indeed, the pioneering work by the van Rij lab on tapiR1 and propiR1 beckons for broader deployment of these type of assays for more mosquito piRNAs and to compare them to miRNAs and siRNAs so we can better judge just how many transgene-matching piRNAs and siRNAs may instigate Piwi or Ago2 silencing. The van Rij lab has recently examined the biochemical silencing capacity of two uniquely abundant *Ae. aegypti* piRNAs that as single sequences can accumulate to nearly miRNA levels in contrast to the general broad swaths of transposon and viral piRNAs. A prominent satellite repeat conserved only amongst culicine mosquitoes has a single-stranded bias in generating just four particularly abundant piRNAs with spacing that defies the proposed configuration of trigger, responder and trailing piRNAs of the ping-pong mechanism [95,97]. The most abundant of the satellite repeat piRNAs is tapiR1, with tens of thousands of reads per million. A second abundant piRNA is propiR1, named as a promiscuous piRNA for being loaded into both PIWI4 and PIWI5 as distinct 30-nt and 27-nt isoforms [96].

Because of the high levels of propiR1 and tapiR1 in *Aedes* Aag2 cells, the luciferase reporters with just single binding sites could be downregulated >10 fold compared to scrambled controls, and only mismatches between targets to the “seed-sequence” of these piRNAs (the 5’ bases at 2nd to 9th position) would affect this strong silencing [95,96]. Curiously, only dsRNA-knockdown of Piwi4 would disable the silencing of reporter genes bearing either propiR1 and tapiR1 target sequences despite some loading of propiR1 into Piwi5. Using antisense oligos to compete out these piRNAs from silencing their endogenous targets, the van Rij lab observed a handful of protein coding genes and one *Ty1-copia* transposon upregulated during tapiR1 inhibition [95], while only a mysterious long noncoding RNA lnc027353 was upregulated by propiR1 inhibition [96]. In the context of *Aedes* development, both tapiR1 and propiR1 are most highly expressed and maternally deposited into the mosquito laid egg and developing embryo [95,96], but their conservation may be restricted to culicine mosquitoes and absent in anopheline mosquito genomes from BLAT searches we conducted [97].

## 6. Are Mosquito piRNA Pathways “Overreacting” to Transgenes?

Various *Drosophila* strains with actively transposing *P*-elements will quickly (in evolutionary timeframes) quench *P*-element expression and mobilization by generating piRNAs antisense to the *P*-element, and may even inhibit proper splicing of introns that when left unspliced renders a truncated and defective *P*-transposase protein [169]. Despite *Drosophila*’s capacity to generate *P*-element piRNAs and other transposon-targeting small RNAs, *Drosophila* transgenesis systems like *P*-elements, *Minos* and *mariner* transposons have been highly successful and stable in transforming *Drosophila* to make stably expressed transgenes [29,170–174]. These transposon transgenesis systems utilize transposase enzymes to bind transposon sequencing within the transgene to mediate transgene DNA integration. One case of *Drosophila* transgene expression being dampened directly by piRNAs is from the endogenous *hsp70* genes that are homologous to certain UAS-gene cassettes with promoter sequences from *hsp70* [175]. An earlier study also showed that an instance of *P*-element transgene silencing in the ovary was reverted by *aubergine* mutations [176]. By and large though, the vast majority of the thousands of *Drosophila* transgenes in transgenic strains are not subjected to piRNA silencing, although multi-copy concatemer integrants would likely generate long dsRNA from inverted copies, and siRNA silencing would ensue [177,178].

With the history of troubled transgenesis attempts in mosquitoes discussed above, we hypothesize that perhaps mosquito transgene constructs that contain transposon sequences like from piggyBac might be triggering an overreactive piRNA response. Our preliminary analysis of sequencing piRNAs from transgenic *An. stephensi* provide a tantalizing support for this hypothesis (Figure 3A). We sequenced total small RNAs from whole adult mosquitoes at ~8 days and ~20 days old from pupation, and we observed a steady increase in both sense and antisense small RNAs corresponding to the GFP reporter gene and plasmid backbone in the pBac transgenesis construct as well as against the Cas9 nuclease. These observations support the possibility that the piRNA pathway may be primed to detect the presence of a transgene. Our future goals are to more broadly sample small RNAs from other transgenic mosquito species and lines to see whether the suppression of the transgene’s expression coincides with the accumulation of either piRNAs or siRNAs presumably generated by the host mosquito line.

To better predict which endogenous mosquito piRNAs can target transgenes, the field will need more complete genomic catalogs of mosquito piRNAs, which has seen the greatest advances in *Aedes* small RNA catalogs [97,103,109,110]. We have also contributed to this effort with a Mosquito Small RNA Genomics (MSRG) pipeline which provides functional annotation and quantitation of mosquito small RNAs into the various types of microRNAs, siRNAs, viral piRNAs and transposon piRNAs [97,179]. The MSRG pipeline’s advantage in annotating mosquito piRNAs is its efficiency in mapping and displaying the piRNAs coming from viruses and transposon consensus elements, but the databases for these entries are still manually curated to reduce redundancy and ambiguity. Examples in Figure 3B show how some insect-specific viruses (ISVs) generate more piRNAs versus siRNAs and vice versa—begging the question of why these differences exist despite both of these ISVs being RNA viruses that replicate via a dsRNA intermediate.

Several mosquito labs studying virus interactions have focused on the mosquito small RNA response to non-retroviral Endogenous Viral Elements (nrEVEs or EVEs) such as flavivirus-like and rhabdovirus-like elements that have integrated into poorly-defined genomic loci of *Aedes* mosquitoes [93,104,105,180–182]. There is high expression of small RNAs in mosquitoes and cell lines against these nrEVEs, such as the primarily antisense piRNAs detected in nearly all *Aedes* aegypti samples against the AEFE1 EVE (Figure 3C). Notably, the pattern of EVE piRNAs is very similar to the extreme antisense patterns of transposon-targeting piRNAs like the example for an *Ae. aegypti Gypsy* element. This pattern may relate to how EVEs can be minor parts of existing transposon segments that make up piRNA clusters [93,110]. But a challenge to this field is tracking the immense flux in these EVE and transposon genomic architectures since cell lines differ from mosquitoes, and EVEs as episomal circular DNAs will likely also fluctuate if the replication and transmission of these episomes is still poorly understood.

Hopefully, more small RNA genomics approaches in mosquitoes will converge on a better understanding of the complete genomic catalogs of mosquito piRNAs. Recently, a study from the van Rij lab deployed chromatin profiling of histone marks, RNA Pol II locations, and chromatin accessibility on *Ae. aegypti* Aag2 cells compared to *Ae. aegypti* tissues to systematically annotate this species’ piRNA clusters [106]. A central proposition from this study is that read-through transcription past the putative 3’ transcription stop of a protein coding gene is a major determinant of a piRNA cluster locus that will also serve as a trap for nrEVEs like flavivirus-like and rhabdovirus-like elements.

However, our new analysis of these *Ae. aegypti* piRNA clusters in Figure 3D suggests a different interpretation if the putative transcription stop elements are mainly based on predicted gene models. We assert that these are genic 3’ UTR piRNA clusters that we have detected previously as a piRNA precursor type conserved across animals [183–186]. Notably, we observe a more prominent retro-transposon trap role in these piRNA clusters rather than an nrEVE trap role, because many *Gypsy* and *BEL* are retroviral-LTR elements that have a biased genomic strand integration pattern at these piRNA clusters. Whether read-through transcription is the basis for piRNA cluster determination is still unclear because juxtaposed to piRNA clusters are other highly expressed gene mRNAs that also generate abundant genic piRNAs but only within the confines of the mRNAs (Figure 3D). Further study may distinguish why one set of genes promote extensive piRNA cluster generation versus being a conventional mRNA being translated into proteins.

It is straightforward to consider that transgenes have all the hallmarks of an invading transposon or virus element, especially since many transgenic approaches in animals are derived from these mobile and infectious entities, from the *P*-element to retroviral vectors. Our preliminary data (Figure 3A) indicate an age-dependent small RNA response to a transgene in *An. stephensi* mosquitoes that we aim to discover in other transgenic mosquito species like *Aedes* and *Culex*. But a key question we need to answer is whether this amount of transgene-targeting small RNAs we see from deep sequencing is sufficient to drive a biochemical silencing effect. How strong is the silencing effect from these transgene-derived siRNAs versus piRNAs versus endogenous miRNAs as the internal calibrator of prototypical gene silencing?

## 7. Are Mosquito Transgenes Triggering Co-Suppression via siRNAs and piRNAs?

In general, the RNAi pathway centered on siRNAs in *Drosophila* is thought to be transient and independent of the Piwi pathway, with few *Drosophila* transgenes known to be co-suppressed by RNAi in a transgenerational manner [23,177,178]. The *Drosophila* piRNA pathway can silence transposons in some short transgenerational situations [124,187–189]. Other than the *hsp70* piRNAs as an example of silencing transgenes in the germline [175], there are relatively few obstacles to getting successful transgene expression in *Drosophila*. The general perception of the biochemical separation of the siRNA and piRNA pathways in most metazoans [17,69,190] may be influenced by also the general observation that RNAi/siRNAs and miRNAs are nearly ubiquitously expressed in the soma and germline while the *Drosophila* and vertebrate Piwi/piRNA pathways are frequently restricted to the gonads [16,68,191].

However, the genetic interactions between siRNA and piRNA pathways have recently been revisited in *Drosophila*. Using various GFP reporter fly strains and siRNA trigger strains, the Aravin lab has asserted that when enough maternal siRNAs are produced in special situations against a target gene, subsequent progeny can morph the siRNA response into a piRNA response for stronger and deeper-inherited silencing [130]. This compelling study though needs additional follow up with other more conventional siRNA triggers against endogenous genes to see if long term germline siRNA expression can more broadly trigger self-propagated piRNA-based gene silencing. An additional inconsistency in this model is that no steady production of piRNAs have been observe in *Drosophila* at the loci that also generate endogenous siRNAs (endo-siRNAs) from satellite-like hairpins and dsRNAs from convergent transcription of two adjacent genes on opposite genomic strands [190,192,193].

Additional studies with novel introductions of invasive transposons into naive *Drosophila* strains akin to a transgene DNA transformation event may support this possible genetic link between siRNAs preceding piRNAs. Earlier studies of a transposon from *Drosophila virilis* called *Penelope* was transplanted to *D. melanogaster* to create dysgenic *Drosophila* crosses, and *Penelope* also first initiated progeny flies to generate siRNAs, but eventually piRNAs were utilized by recovering fly progeny to complete *Penelope* silencing [132,194]. More recently, the Kofler lab put mobile *P*-elements in *Drosophila simulans* populations to also trigger gonadal dysgenesis, but two populations would then rebound their fertility by first generating siRNAs against the *P*-element that in later generations become piRNAs that enforce stable transposon silencing [195]. However, a third population in Kofler study at first initiated a strong siRNA response, but then there was still a failure to establish a stable piRNA response, suggesting that siRNA generation is not a guarantee for an eventual piRNA response.

Potential interactions between mosquito siRNAs and piRNAs has not been as extensively explored yet in mosquitoes, though mosquito mutants and knockdowns of *Dcr2* and *Ago2*, main components making and holding siRNAs, respectively, have indicated that virus replication is potentiated when RNAi activity is lost and viral siRNAs are generated alongside viral piRNAs [196–201]. Broader genomics surveys of *Aedes* small RNAs further show that transposon and virus can be targeted simultaneously by both siRNAs and piRNAs, but piRNAs clearly dominate as a much higher targeting proportion compared to siRNAs [97,103,110,180,202]. As of yet, the types of large hairpins and overlapping gene transcripts generating endo-siRNAs in *Drosophila* [193] have not been easily detected in mosquito genomes.

## 8. Can We Mask Transgenes from Mosquito RNAi Responses?

With exemplar piRNAs like tapiR1 and propiR1 that can strongly silence targets during mosquito embryogenesis [95,96], we can predict that genes like Piwi4 may be as necessary for viability as the *Ago1* gene that bind miRNAs. If these piRNAs and other critical piRNAs are hypothetically targeting transgenes for silencing, it may not be feasible to inhibit these specific piRNAs in transgenic mosquitoes because embryonic development could be adversely affected.

**Figure 4.**
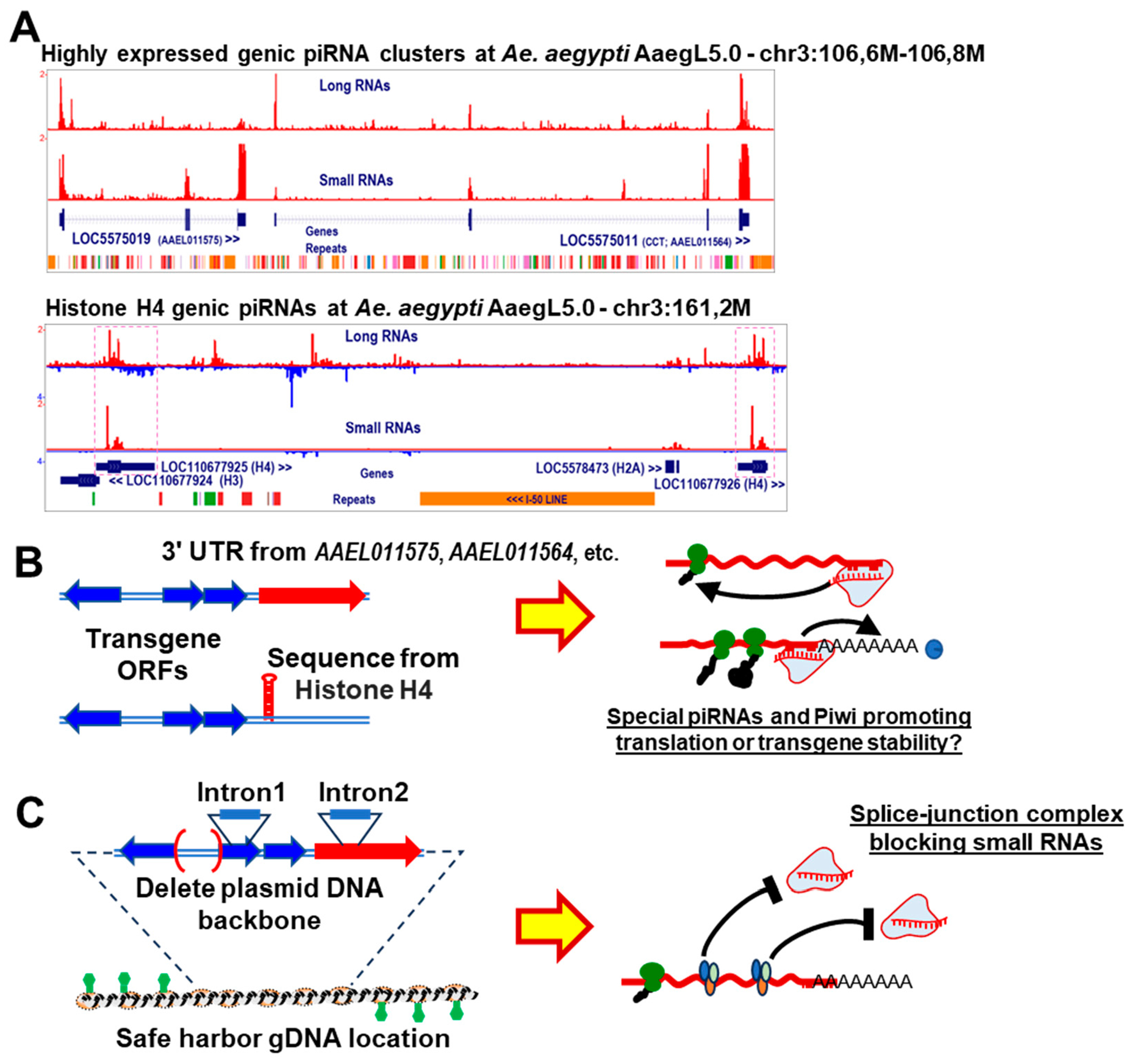
Most endogenous mosquito piRNAs are designed for gene silencing, but transgene DNAs could be better engineered to have activating piRNAs or safe harbor DNA features to avoid small RNA silencing. (**A**) Browser plots showing abundant genic piRNA production from two types of protein coding genes in *Aedes aegypti*. The upper panel shows two adjacent protein coding genes generating abundant mRNAs as well as genic piRNAs predominantly from the gene exons. The lower panel shows that histone H4 genes marked by the pink dash boxes are often generating abundant small RNAs with patterns the differ from the other histone mRNAs being transcribed. (**B**) Diagram speculating that utilizing these genic-piRNA elements may demarcate transgenes as appearing “less-foreign” to the mosquito host genome. (**C**) Alternatively, adding introns to transgene DNA constructs that currently lack them and removing plasmid backbone DNA could license these transgenes and prevent small RNA silencing.

Therefore, we speculate that other types of genic piRNA sequences from genes that are highly expressed may act as a molecular “decoy” within the mosquito’s shuffling of small RNAs into multiple Ago and Piwi proteins in the soma. For example, there are a multitude of protein coding genes in *Aedes* that generate a slew of genic piRNAs as well as stable accumulation of the mRNA that is likely being translated into protein (Figure 4A). These somatic genic piRNA profiles are less biased for the 3’ UTR compared to the prototypical germline-restricted genic piRNA clusters [183,184] or even the other transposon-trapping mosquito piRNA clusters which could be either a form of read-through transcription or an extended 3’ UTR genic piRNA cluster [96]. Although the 3’ UTR genic piRNAs from the *Drosophila Traffic Jam* piRNA cluster can direct *Piwi*-mediated silencing of a reporter gene with antisense *Tj*-3’ UTR sequences [160], we are re-evaluating whether *Piwi* itself would affect *Tj* expression levels as alluded to in other studies [184,185].

The expansion of seven Piwi genes in culicine mosquitoes is reminiscent of the diversification 23 Ago genes in the nematode *Caenorhabditis elegans* that include nematode-specialized piRNAs that are also called 21U small RNAs as well as several types of 22G and 26G endo-siRNAs [15,203]. Whereas most *C. elegans* small RNAs inhibit gene expression, there are 22G small RNAs bound by the CSR-1 Argonaute protein that promote gene expression by modulating or competing against the other silencing Argonaute/siRNA complexes including *C. elegans* piRNAs [15,203]. The anti-silencing activity of CSR-1 is thought to license self-transcripts for expression while distinguishing from invading transposons and viruses prominently targeted by the PIWI and WAGO siRNAs [161,204–209]. Most of the CSR-1 targets are germline-expressed genes in both oocytes and sperm precursor cells, and indeed an *oma-1::gfp* transgene can be ‘licensed’ for GFP expression in specific worms when the transgene engages in loading transgene small RNAs into CSR-1 [210,211].

If one of the seven Piwi genes in culicine mosquitoes shares a gene enhancing role like CSR-1, perhaps the sequence elements of highly expressed mosquito genes that generate abundant genic piRNAs could be inserted into transgenes to also license this DNA (Figure 4B). For example, the 3’ UTRs of highly expressed mosquito genes like *AAEL011575* or *AAEL011564* that also generate abundant piRNAs could confer special genic piRNA products to license transgene expression stability as well as potentially unwanted regulatory elements in those genes’ 3’ UTR. For a very short licensing sequence, perhaps the histone H4 sequence could be explored since a significant amount of mosquito genic piRNAs derive from this gene [91].

The function of histone H4 piRNAs is still mysterious, but a recent study posited that *Aedes* infected with Humaita Tubiacanga Virus (HTV), Phasi-Charoen-Like Virus (PCLV), and dengue virus (DENV) caused greater histone H4 expression than just DENV infection alone [212]. Since HTV, PCLV and DENV infections of mosquitoes and cells generates abundant viral piRNAs [97,101,102,112,200,212], perhaps the triple load of viruses ramps up mosquito resistance responses feeding back to H4 and possibly H4 piRNA levels. Connecting back to nematodes’ RNAi pathways, mutations disrupting *C. elegans* piRNAs can also affect histone mRNA regulation [209,213–215]. Perhaps a common thread for transgene’s expression stability being modulated by invertebrate RNAi pathways is a nexus of regulation of histone gene expression.

This speculation of finding activating Piwi/piRNA complexes could be more challenging to extend to anopheline mosquitoes which like *Drosophila*, only have three Piwi gene members, but they do also have several genic piRNA clusters [97]. With transgene expression stability a problem in *Anopheles* like *An. stephensi* and the detection of transgene-targeting piRNAs (Figure 3A), we should further study insect virus infectious clones as transgenesis systems to determine during time courses when after virus infection the reporter transgene becomes silenced. For example, a densovirus infectious clone bearing a GFP transgene could transduce *An. gambiae* Mos55 cells very successfully generate fluorescent cells [216,217], but with time the fluorescence was lost in the transduced cells. Indeed, the Mos55 cells no longer expressing the GFP transgene within the densovirus was now generating viral piRNAs antisense to densovirus [97], but a second distinct isolate of Mos55 cells was also infected with densovirus but lacked densoviral piRNAs. If we can figure out how to modulate insect virus infectious clones to be highly infectious of mosquitoes yet can avoid generating viral piRNAs, these mutant viruses may side step unstable expression of mosquito transgenes.

Lastly, we also need to discover the mosquito genome loci where inserted transgenes either trigger undesired RNAi silencing or are stably expressed when the DNA location is a “safe-harbor” with favorable chromatin conformations. Our observations that many highly expressed mosquito protein coding genes can still lead to high genic piRNA production (but no siRNA production), could suggest that there are many DNAs with favorable chromatin conformations for transgenes that could still lead to some small RNA production. Perhaps ATACseq and genome-wide histone marks profiles for mosquitoes like in [106] will be a great resource to datamine for such safe-harbor loci. Alternatively, genome-wide CRISPR-mediated sgRNA libraries being developed for mosquito genomes such as in [218], can be expanded for testing long-term stable transgene expression in mosquito cells that then may translate to transgenic mosquitoes [219].

The concept of genomic DNA safe harbors that can shield transgenes from RNAi silencing has been demonstrated in *C. elegans* where non-coding DNA carrying 10-base pair periodic An/Tn-clusters (PATCs) enable stable transgene expression in the nematode’s germline [220]. These PATCs DNA sequences found in germline genes can override piRNA-complementary sequences from being silenced by piRNAs [221]. Further optimization of transgene DNAs inserted into these PATCs in *C. elegans* also include adding introns and removing plasmid DNA backbone sequences [222], since transgenes lacking introns and containing plasmid sequences have been observed to trigger RNAi silencing in *C. elegans* [223,224]. We can take a page from the worm to add these features to mosquito transgenes (Figure 4C) to possibly address our tribulations with transgene expression instability. It has not escaped our attention that several endogenous small RNAs in *An. stephensi* primarily map to the plasmid backbone sequence (Figure 3A).

As climate change and globalization trends lead to greater co-habitation of mosquitoes in densely human-populated areas, we will face increased risk of human infectious diseases vectored by mosquitoes. Transgenic mosquitoes should theoretically be receptive to all our modern genetic and genomic innovations, but over 40 years of mosquito transgene tribulations should not deter researchers. By recognizing the value of cross-species model organism comparisons, we propose supporting more studies to leverage our knowledge of *Drosophila* RNAi pathways to unlock the secrets within mosquitoes Piwi/piRNA pathways.

## Data Availability

All the relevant data are provided in the manuscript.
